# Editorial: Porcine reproductive and respiratory syndrome virus - animal virology, immunology, and pathogenesis

**DOI:** 10.3389/fimmu.2023.1194386

**Published:** 2023-04-20

**Authors:** Chunhe Guo, Xiaohong Liu

**Affiliations:** ^1^ State Key Laboratory for Animal Disease Control and Prevention, Key Laboratory of Zoonosis Prevention and Control of Guangdong Province, College of Veterinary Medicine, South China Agricultural University, Guangzhou, Guangdong, China; ^2^ Guangdong Laboratory for Lingnan Modern Agriculture, Guangzhou, Guangdong, China; ^3^ State Key Laboratory of Biocontrol, School of Life Sciences, Sun Yat-sen University, Guangzhou, Guangdong, China

**Keywords:** porcine reproductive and respiratory syndrome virus, control, immunology, pathogenesis, vaccine

Porcine reproductive and respiratory syndrome (PRRS) is one of the most important pig diseases causing huge economic losses worldwide. The causative agent, PRRS virus (PRRSV), is an enveloped, single-stranded, positive-sense RNA virus which is classified into the genus *Betaarterivirus*, subfamily *Variarterivirinae*, family *Arteriviridae* along with equine arteritis virus (EAV), lactate dehydrogenase-elevating virus of mice (LDV), and simian hemorrhagic fever virus (SHFV). Its genome is about 15 kb in length and contains at least 11 open reading frames (ORFs) with 5´ cap and 3´ polyadenylated tail (1–3). The nonstructural proteins (nsp1-12), owning the functions of protease, replicase and regulation of host cell gene expression and responsible for the synthesis of viral RNA, are encoded by ORF1a and ORF1b which occupy approximately two-thirds of the genome (4). The structural proteins including glycoprotein 2 (GP2), GP3, GP4, GP5, envelope protein (E), matrix protein (M), and nucleocapsid protein (N), expressed by a series of subgenomic RNAs, are encoded by ORFs 2-7 at the 3´ terminus of the genome (5). Due to the lack of proofreading ability of PRRSV RNA-dependent RNA polymerase (RdRp), the viral genome is highly susceptible to mutation and recombination, which causes the emergence of novel PRRSV isolates worldwide (6). At present, PRRSV can be divided into two species: PRRSV-1 (European genotype, Betaarterivirus suid 1) and PRRSV-2 (North American genotype; Betaarterivirus suid 2). Both species show high genetic diversity and share approximately 60% nucleotide sequence identity, and each one can be further divided into several clades, substrains or lineages. In China, the dominant strains are PRRSV-2 and the outbreaks of highly pathogenic variants lead to concerns in the pig industry (7). PRRSV infection causes severe reproductive failure in sows and respiratory disease in pigs of all ages, which often leads to secondary bacterial infections (such as Haemophilus parasuis and Streptococcus suis) with greater clinical manifestations and higher mortality (8).

PRRSV has an extremely narrow tropism for cells *in vivo* and *in vitro* and porcine alveolar macrophages (PAMs) are the main cells for the study of PRRSV pathogenesis *in vitro* (9, 10). Therefore, the virus is known to specifically infect pigs and other species are not susceptible to it. Since PRRSV mainly infects PAMs and destroys the immune system, the virus causes cytokine storm and immunosuppression thus inducing poor innate and acquired immune responses in pigs. In addition, due to the antibody-dependent enhancement (ADE) induced by the virus, some antibodies such as non-neutralizing antibodies or antibodies at sub-neutralizing levels might have undesirable effects to promote virus invasion and exacerbate virus-induced damage (11–14). In PRRSV-infected animals, the virus leads to persistent infection resulting in long viremia lasting for more than four weeks. During persistent infection, although pigs may show no clinical symptoms the virus is capable of maintaining replication in tonsils and lymph nodes for several months, keeping shedding in the environment (15).

Collectively, due to the above six characteristics of PRRSV, PRRS prevention and control remain a worldwide issue. Commercial vaccines including modified-live vaccines (MLV) and inactivated vaccines are available but provide only partial or no protection against PRRSV infection (16). Currently, vaccines have many flaws such as recombination with field strains, potential risk of virulence reversion, and destroying or interfering with the immune responses (17). New strategies are required to mitigate the devastating consequences of this disease. The molecular mechanisms of PRRSV pathogenesis and pathogen-host interactions are not fully clear.

The humoral immune responses play important roles in the clearance of pathogens. Kick et al. investigated the local and systemic humoral immune responses in pigs inoculated with one of three types of PRRSV-2 (one MLV vaccine strain and two lineage 1 isolates) *via* quantifying local immunoglobulin A (IgA) and systemic IgG and homologous and heterologous neutralizing antibodies (NAs). They found that the local IgA response closely follows viral shedding and the systemic IgG response also starts shortly after viremia but keeps high until the end of this study. Additionally, the two lineage 1 strains used in this study almost cannot induce NAs within 1-2 weeks after infection and the antibodies show only a shortly delayed within lineage cross-reactivity. The study improves our understanding of the relationships between local and systemic infections and the humoral immune responses induced by PRRSV or MLV vaccination and suggests a potential to develop novel vaccines against PRRSV infection using these strains. Previous studies have shown that a small proportion of serum containing broadly reactive NAs is capable of recognizing and neutralizing heterologous strains (18). Nevertheless, it remains unknown whether the cross-reactivity in seroneutralization experiments carried out *in vitro* correlates with the protection *in vivo*. Therefore, Martı´nez-Lobo et al. explored whether the strains that induce broadly reactive NAs *in vitro* have the ability to confer better protection *in vivo* after exposure to heterologous isolates than the viruses which induce strain-specific NAs. The data demonstrated that PRRSV-1 strains differ in their capacity to induce cross-reactive NAs and confer protection against heterologous reinfections. This research suggests that broadly reactive NAs play a vital role in the protection against heterologous infections.

The understanding of PRRSV epitopes related to protection contributes to the development of optimized PRRSV vaccines. Young et al. stated that exposure to various PRRSV isolates enhances and improves the possibility of the generation of broadly NAs. As a result, a reverse vaccinology approach is taken, and memory B cells from two pigs, which are exposed to both the PRRSV vaccine and field isolate several times and whose serum neutralizes a broad range of PRRSV isolates, are immortalized. The results showed that antibodies from the above B cells have the ability to bind all the PRRSV-2 isolates used in the study but neither of the PRRSV-1 isolates. Further, the antibodies against the GP5 protein which is considered to have dominant neutralizing epitopes are found to be highly abundant as four out of five B cells are GP5-specific. However, only one antibody is determined to neutralize homologous but not heterologous strains. This research confirms that the GP5 protein of PRRSV contains neutralizing epitopes and broad binding, non-neutralizing antibodies may precede those that are broadly neutralizing. Future-designed vaccines that possess diverse PRRSV strains or genetically distinct epitopes are more effective in cross-neutralizing a broad range of PRRSV isolates. The thymus, a primary lymphoid organ, plays a key role in T-cell development and maturation. It is reported that the highly pathogenic PRRSV strains such as the Lena strain show a higher thymus tropism than low virulent isolates and cause severe thymus atrophy thus affecting immune responses in pigs. To investigate the role of immune checkpoints in the thymus of piglets upon PRRSV infection, Ruedas-Torres et al. outlined the expression of selected immune checkpoints (PD1/PDL1, CTLA4, TIM3, LAG3, CD200R1 and IDO1) in the thymus of animals during the early stage of two different PRRSV strains infection. The checkpoints of PD1/PDL1, CTLA4, TIM3, LAG3 and IDO1 are significantly up-regulated in the thymus of infected pigs, especially in those challenged with the virulent isolate. The up-regulation may be involved in disease progression and viral load and shedding. This study contributes to the interpretation that why the highly pathogenic PRRSV strains cause more severe pathological and tissue damage than the low virulent isolates. PRRSV infection usually results in a delayed response of the production of NAs, indicating that the virus may suppress or deceive the immune system. The interaction of the virus with dendritic cells (DCs), specialized in antigen presentation, could be one of the molecular mechanisms of deception. Li and Mateu explored the influence of a moderately virulent PRRSV strain on the maturation, production of cytokine, and antigen presentation capacity of the three DC populations: conventional DCs (cDC1 and cDC2) and CD14^+^ DCs. They found that exposure to PRRSV-1 does not induce the maturation of cDC1, cDC2, or CD14^+^ DCs. However, it may affect toll-like receptor-associated responses except for cDC1, which may interfere with the development of acquired immune responses during PRRSV infection. The molecular mechanisms that why PRRSV infection leads to a poor and delayed immune response remain to be further addressed. MLV have been widely used in pig farms and can indeed reduce clinical symptoms and economic losses mainly in the context of PRRS outbreaks. However, these MLV have many side effects and the reasons for this remain unclear. Bocard et al. revealed the relationships between innate and adaptive immune responses upon PRRSV infection or vaccination. Intramuscular MLV vaccination in pigs often leads to an opposite regulation of blood transcriptional modules (BTMs) when compared to intranasal inoculation with highly pathogenic field isolates. This study may contribute to the explanation of the complexity of the innate and acquired immune responses against PRRSV infection and suggests a fundamentally different immune response to less immunogenic MLV compared with field isolates. In the design of safer and more efficient vaccines, it is a crucial challenge to address the balance between immunogenicity and attenuation of the field viruses. The cell cycle, consisting of gap 1 (G1), synthesis (S), gap 2 (G2), and mitosis (M), is a complex physiological process involved in the cell genome replication and growth as well as cell division. The relationships between PRRSV and cell cycle and how the virus exploits cell cycle to promote its proliferation are largely unknown. Wen et al. reported that PRRSV infection results in Marc-145 cells entry into S stage, therefore contributing to virus proliferation. Mechanistically, PRRSV nsp11 is capable of degrading p21 thus leading to the promotion of cells entry into S phase. Moreover, the endoribonuclease activity but not the deubiquitination activity of viral nsp11 mediates the degradation of p21. The study lays the foundation for further understanding of pathogen-host interactions.

It is critical to address PRRSV pathogenesis and the molecular mechanisms of virus-host interactions, which is a key prerequisite and basis for preventing and controlling the disease. Since PRRSV is an immunosuppressive pathogen, the development of novel vaccines that induce higher NAs and are safer (especially vaccine virulence reversion) is a major challenge. PROteolysis-TArgeting Chimeras (PROTACs), consisting of three structures: a ligand for binding with the protein of interest (POI), a ligand for recruiting an E3 ligase and a linker, have been developed to exploit and hijack the ubiquitin-proteasome system (UPS) of the host cell to specifically mediate the highly selective degradation of POIs (**Figure 1A**). Until now, this technique has achieved remarkable results in the fields of cancer treatment and overcoming drug resistance. PROTACs also show huge potential in the development of new vaccines. Si et al. engineered a conditionally removable proteasome-targeting domain (PTD) onto the matrix fragment of influenza A virus (19). The viral matrix protein is specifically destroyed by the host UPS, thereby the proliferation of the virus is dramatically attenuated while maintaining the ability to induce broad and robust humoral and cellular immune responses. The novel PROTAC influenza virus vaccine can elicit efficiently protection against homologous and heterologous challenges. As a result, the PROTACs technique also can be used in the production of MLV against other pathogens such as PRRSV. As shown in **Figure 1A**, the ligand for recruiting an E3 ligase is specifically inserted into a certain protein of PRRSV (designated as protein A), which is then degraded by host cellular UPS to attenuate the virus replication (replication-deficient). This PRRSV vaccine candidate is very promising for clinical applications since it is indeed safer than current commercial MLV used in pig farms. Although a number of PRRSV receptors and the functions of the viruse-encoded proteins have been extensively studied, little remains clear concerning the requirements of host factor of the virus. The identification of crucial host cellular factors involved in the different phases of the virus life cycle contributes to the development of countermeasures and increases preparedness for potential future PRRS outbreaks. It represents the objective of intense efforts. CRISPR (Clustered Regularly Interspaced Short Palindromic Repeats) screens have become a powerful source of biological discovery as they offer a powerful platform for screening in diverse fields such as drug development and identification of potential host factors essential for virus replication (20, 21). It is reported that the CRISPR/Cas9-based screen approach using lentiviral single-guide RNA libraries enables the pooled loss of function screens with high specificity and sensitivity (22–26). This strategy to investigate pathogen-host interactions is to globally disrupt individual genes of the whole host cellular genome and identify those whose disruption leads to resistance to viral invasion. Based on it, several genome-wide knock-out CRISPR screens for the identification of the regulators of SARS-Coronavirus-2 or other viruses especially the potential receptors have been studied (27–30). Similarly, as shown in **Figure 1B**, we can perform a genome-wide CRISPR/Cas9 loss of function screening to identify potential host proteins required for PRRSV infection. After screen, the key genes promoting virus infection need to be further confirmed which could be used as targets for the development of antiviral drugs disrupting the synthesis of host factor mRNA or protein. More importantly, several key factors like CD163 receptor (31; Guo et al.), essential for the virus entry into host cells, have great potential for the breading of disease-resistant pigs by CRISPR/Cas9 technology.

**Figure 1:**
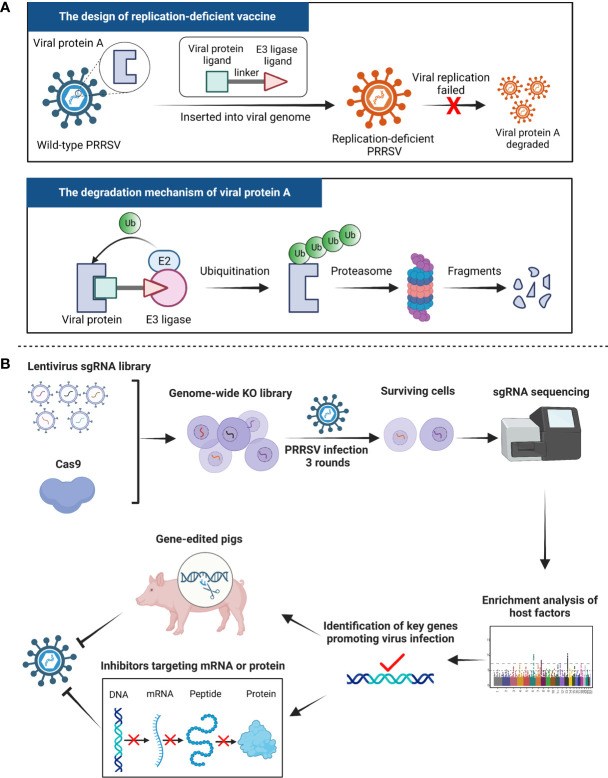
Ideas and strategies for PRRSV prevention and control in future. **(A)** PROteolysis-TArgeting Chimeras (PROTACs), consisting of three structures: a ligand for binding with the protein of interest (POI), a ligand for recruiting an E3 ligase and a linker, have been developed to exploit and hijack host cellular ubiquitin-proteasome system (UPS) to specifically mediate the highly selective degradation of POIs. We can use this technique to develop a novel PRRSV replication-deficient vaccine candidate. A certain protein of the virus (namely protein A) is specifically degraded by the host UPS thus the production of progeny viruses fails, suggesting that the vaccine candidate is indeed safer than current modified live vaccines. **(B)** A genome-wide CRISPR/Cas9 screening has great potential for the study of PRRSV-host interactions and the identification of the key regulators mediating virus entry and infection. The key host genes could be used as targets for the development of antivirals and the breeding of disease-resistant pigs.

## Author contributions

All authors listed have made a substantial, direct, and intellectual contribution to the work, and approved it for publication.
